# Genomic Expression Libraries for the Identification of Cross-Reactive Orthopoxvirus Antigens

**DOI:** 10.1371/journal.pone.0021950

**Published:** 2011-07-14

**Authors:** Lilija Miller, Marco Richter, Christoph Hapke, Daniel Stern, Andreas Nitsche

**Affiliations:** Robert Koch-Institut, Centre for Biological Security 1, Berlin, Germany; University of Georgia, United States of America

## Abstract

Increasing numbers of human cowpox virus infections that are being observed and that particularly affect young non-vaccinated persons have renewed interest in this zoonotic disease. Usually causing a self-limiting local infection, human cowpox can in fact be fatal for immunocompromised individuals. Conventional smallpox vaccination presumably protects an individual from infections with other *Orthopoxviruses*, including cowpox virus. However, available live vaccines are causing severe adverse reactions especially in individuals with impaired immunity. Because of a decrease in protective immunity against *Orthopoxviruses* and a coincident increase in the proportion of immunodeficient individuals in today's population, safer vaccines need to be developed. Recombinant subunit vaccines containing cross-reactive antigens are promising candidates, which avoid the application of infectious virus. However, subunit vaccines should contain carefully selected antigens to confer a solid cross-protection against different *Orthopoxvirus* species. Little is known about the cross-reactivity of antibodies elicited to cowpox virus proteins. Here, we first identified 21 immunogenic proteins of cowpox and vaccinia virus by serological screenings of genomic *Orthopoxvirus* expression libraries. Screenings were performed using sera from vaccinated humans and animals as well as clinical sera from patients and animals with a naturally acquired cowpox virus infection. We further analyzed the cross-reactivity of the identified immunogenic proteins. Out of 21 identified proteins 16 were found to be cross-reactive between cowpox and vaccinia virus. The presented findings provide important indications for the design of new-generation recombinant subunit vaccines.

## Introduction

The genus *Orthopoxvirus* (OPV) from the family *Poxviridae* contains complex viruses which replicate entirely in the cytoplasm of the infected cell [1], [2]. Their linear double-stranded DNA genome of up to 220 kbp [1] contains no introns and encodes more than 200 open reading frames (ORFs) [3]. The genus is best known for two of its most prominent species: vaccinia virus (VACV) and variola virus (VARV). Interestingly, VACV was used to eradicate VARV, the causative agent of smallpox, through a worldwide vaccination campaign [4], [5]. This was possible due to an antigenic relationship between members of OPVs. An earlier infection with one of these members provides some protection against subsequent infections with the others [6]. Nevertheless, the declaration of the successful eradication of smallpox in 1980 [7] led to the discontinuation of the routine smallpox vaccination [8] due to the risk of rare but severe adverse reactions [9], [10]. Other human-pathogenic OPV members include monkeypox virus and cowpox virus (CPXV) [1], the latter having the largest genome of all OPVs [11].

CPXV is prevalent in Western Eurasia and has an extremely broad host range [4], [12]. Human cowpox is a zoonotic disease, usually transmitted by cats, which mostly causes self-limiting local infections [13]. However, severe clinical courses resulting in prolonged treatment and scarring have been described [13], [14]. Furthermore, a case of generalized, fatal CPXV infection in an immunocompromised patient with a life-long history of atopic dermatitis has been reported [15]. Human cowpox particularly affects young people [16], indicating that the lack of smallpox vaccination may render today's population more susceptible to OPV infections including cowpox [17], [18]. At the same time there is no approved effective antiviral treatment available, and conventional smallpox vaccines recently administered can cause rare but severe adverse reactions [5], notably affecting immunodeficient individuals and those with atopic dermatitis [19], [20]. Unfortunately, this group is already at a higher risk to develop OPV infections with a severe clinical course. Therefore, there is a pressing need for the development and approval of safer and more effective vaccines [17].

Recombinant subunit-based vaccines represent possible alternatives to the conventional smallpox vaccines [21]. To develop these vaccines, it is necessary to identify those antigens inducing the most effective immune response. Several antigenic VACV proteins and combinations thereof have been successfully tested in animal models [22]–[24]. However, beside individual immune responses, the genetic diversity of poxviruses has to be taken into account when designing subunit vaccines [25]. It has long been known that VACV and CPXV show immunological similarities as well as differences [26]–[28]. However, as yet little is known about the cross-reactive CPXV antigens. The inclusion of antigens that are cross-reactive to several OPV species in subunit vaccines could potentially help confer a resilient immune response [21] and prepare more effective subunit vaccines.

In this study, we first constructed and evaluated four different genomic bacteriophage λ-based expression libraries (EL) containing the VACV and CPXV genomes. The EL were then serologically screened using sera from VACV-immunized humans and animals as well as clinical serum samples from CPXV-infected humans and animals. Through these screenings we were able to identify 21 immunogenic proteins of CPXV and VACV. The identified proteins show diverse functions and a genome-wide distribution, with surface proteins as well as non-surface (structure) proteins being present. By analyzing the whole set of antigens, we found 16 out of 21 proteins to be cross-reactive between CPXV and VACV. Six of these 16 cross-reactive proteins are also perfectly conserved among all OPVs. Seven cross-reactive proteins are proposed to be tested as components of subunit vaccines. We therefore describe a low-priced approach to antigen discovery which is especially well suited for investigation of large DNA viruses. The approach described is independent of a prior knowledge of antibody targets. Software-based ORF prediction and primer design are thus not required. The method described is therefore suitable for the identification of cross-reactive proteins shared by further clinically relevant OPV species beside those of VACV and CPXV. The integration of clinical serum samples in the screening experiments in addition to sera obtained from immunized individuals further provides a more authentic situation, allowing the identification of the most antigenic proteins. These proteins might be especially well suited for inclusion in subunit vaccines.

## Results

### Construction and validation of genomic OPV expression libraries

The data presented here were derived from serological screenings of four genomic OPV EL varying in the genome species expressed and insert size. The genomic EL were constructed by cloning fragments of genomic OPV DNA into a modified bacteriophage λ-based vector (ZAP Express). This vector can accommodate DNA fragments with a length of up to 12 kb. The cloned DNA fragments can be excised out of the phage in the form of the kanamycin-resistant pBK-CMV phagemid vector, which allows to characterize insert DNA in a plasmid system.

Two of the EL described were constructed using partially digested CPXV genomic DNA of strain GuWi and designated according to insert size: *EL-CPXV-0.2k-0.7k* and *EL-CPXV-0.2k-3k*. Additionally, two EL containing the VACV genome strain New York City Board of Health (NYCBH) were constructed and designated *EL-VACV-3k-12k* and *EL-VACV-0.2k-0.7k*. Exemplary, *EL-CPXV-0.2k-3k* is an EL containing 0.2–3 kb inserts of a partially digested CPXV genome. The recombinant titer of the constructed EL ranged from 3×10^6^ to 2×10^9^ plaque forming units per milliliter.

**Figure 1:**
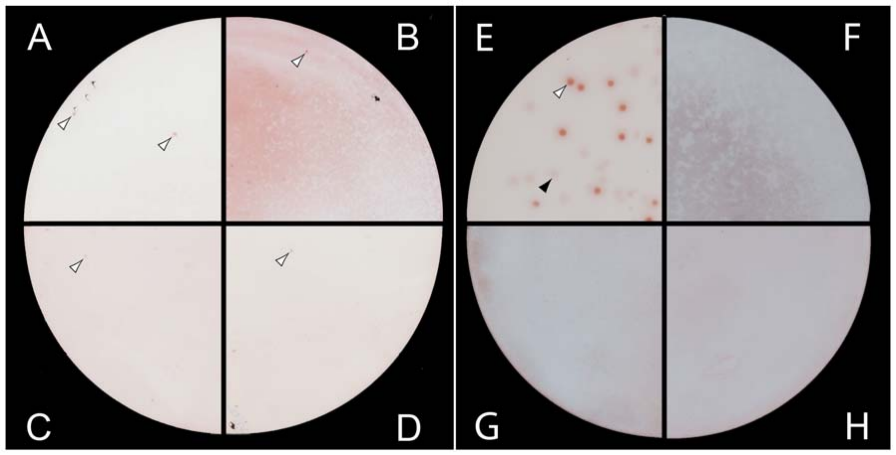
Validation of constructed genomic expression libraries. For a validation of their complexity the constructed genomic EL were serologically screened with polyclonal and monoclonal antibodies of known specificities. Immunopositive signals are exemplary indicated through white arrows for the following screening combinations: (A) *EL-CPXV-0.2k-0.7k* with polyclonal goat anti-rA27 serum, (B) *EL-CPXV-0.2k-3k* with monoclonal mouse anti-rA27 antibody, (C) *EL-VACV-3k-12k* with goat anti-rA27, and (D) *EL-VACV-0.2k-0.7k* with goat anti-rA27. Further evaluation was performed by immunoscreening a genomic CPXV EL with different sera and controls: (E) Immunoscreening of *EL-CPXV-0.2k-3k* using serum from a VACV-immunized rabbit. The white arrow points to an immunopositive plaque, the black arrow to an immunonegative plaque. (F) Goat anti-rabbit IgG conjugate alone without primary antibody. (G) Human serum from a poxvirus-naive person. (H) Human poxvirus-naive anti-dengue virus serum.

For the validation process recombinant plaques were randomly picked and the insert DNA sequenced. The obtained sequences were aligned with an appropriate OPV genome. Figure S1 schematically shows the distribution of the sequences on an OPV genome for *EL-VACV-3k-12k* (A) and *EL-CPXV-0.2k-0.7k* (B). For further validation of their complexity the constructed libraries were screened with monoclonal (anti-rA27, anti-CPXV 3D11) and polyclonal (goat anti-rA27) antibodies. First, the libraries were screened with the monoclonal anti-CPXV 3D11 antibody. This antibody was generated against native CPXV particles [29]. Through library screenings, immunopositive signals for the expected antigen could be identified (Figure S2), as confirmed by sequencing. The anti-rA27 antibodies were first tested in an ELISA system. Here they were shown to recognize virus particles as well as the recombinant protein expressed in *E. coli* that was used to immunize goats and mice (Figure S3). Through library screenings, positive signals for the expected antigen were identified for all tested antibodies (Figure 1A–D), as confirmed by sequencing.

To show the functionality of the screening system, several controls were performed (Figure 1E–H). For the identification of the most antigenic proteins and a maximum reduction of the background signal all sera were diluted 1/200 to 1/1000. All human sera were preincubated with *E. coli* lysate to remove anti-*E. coli* antibodies that might potentially be present. Thus, after reducing the background signal, a clear differentiation between positive and negative signals was possible (Figure 1E). No positive signals resulted from an incubation with the appropriate secondary antibody alone (Figure 1F). In addition, for a demonstration of selective specificity, the EL were screened using poxvirus-naive serum (Figure 1G) as well as a poxvirus-naive serum with a high anti-dengue virus titer (Figure 1H). No positive signals were seen in the control screenings.

### Identification of immunogenic VACV proteins

For the construction of EL, genomic OPV DNA was partially digested with the Bsp143I restriction enzyme and cloned into the ZAP Express vector. Through serological screenings immunoreactive phage clones were identified and the insert DNA sequenced. The DNA sequences obtained were aligned with an appropriate reference OPV genome for the identification of the encoded protein(s). For convenience, the genes encoding the identified immunogenic proteins are indicated according to the established convention of naming VACV genes or ORFs. This consists of using the HindIII restriction endonuclease DNA fragment letter (A-P), followed by the ORF number within the fragment, and L or R, depending on the direction of the ORF [2]. When inserts encode more than one protein, no precise identification of the antigenic protein is possible. In that case, the position of the identified genomic region is indicated through the respective HindIII fragment letter.

The constructed genomic EL were screened with anti-OPV sera to scan the humoral immune response from vaccinated or infected humans and animals. EL containing the VACV genome were used to identify immunoreactive proteins of VACV. This should serve as a proof of principle for the functionality of the EL prior to the identification of CPXV-reactive proteins. The serological screening of *EL-VACV-3k-12k* using sera from immunized humans (Vaccinia immune globulin, VIG) and rabbits as well as clinical human sera resulted in the identification of several immunoreactive plaques. After sequencing of the insert DNA, immunodominant genome regions encoding several proteins could be identified. One of the most frequently detected genomic regions was in the HindIII A fragment, which almost always included the gene WR148, the VACV orthologue of the cowpox A-type inclusion protein (A25). Proteins encoded by HindIII fragments D and C/B could also be identified.

The screening of *EL-VACV-3k-12k* did not result in the precise attribution of immunoreactive plaques to individual proteins, owing to the fact that the relatively long inserts often encode more than one protein. To circumvent this problem, *EL-VACV-0.2k-0.7k* was constructed. The serological screening of this EL using rabbit antiserum resulted in the identification of the immunoreactive proteins A18 (DNA helicase), A47 (hypothetical protein), B2 (function unknown), and E3 (double-stranded RNA-binding protein) (Table 1).

**Table 1:** CPXV and VACV antigens identified by screening of genomic expression libraries.

VACV-Cop homolog	Gene name	Accession numbers	EL^a^	EL^b^	EL^c^	Function	IEDB hits^d^
**Rabbits** e							
*A18R*	*WR-138* f	*AAO89417*	x			*DNA helicase*	+
*A47L*	*WR-173*	*AAO89452*	x			*Hypothetical protein*	+
*B2R*	*WR-184*	*AAO89463*	x			*Unknown*	+
A3L	CPXV135^g^	AAM13576		x		P4b precursor	+
A25L	CPXV158	AAM13600		x	x	A-type inclusion body protein	+
A48R	CPXV186	AAM13626			x	Thymidylate kinase	+
A53R	CPXV191	AAM13631		x		Tumor necrosis factor receptor (CrmC)	−
B10R	CPXV204	AAM13643			x	Kelch-like protein	+
B22R	CPXV219	AAM13657			x	Surface glycoprotein	−
C10L	CPXV033	AAM13480		x		Hypothetical protein	+
C23L	CPXV003	AAM76298		x		Chemokine-binding protein	−
E2L	CPXV068	AAM13513			x	Hypothetical protein	+
E3L	*WR-059*CPXV069	AAO89338AAM13514	x	x		Double-stranded RNA-binding protein	+
E9L	CPXV075	AAM13520			x	DNA polymerase	+
H6R	CPXV115	AAM13558			x	DNA Topoisomerase type I	+
M1L	CPXV039	AAM13486		x		Ankyrin-like protein	+
**Humans** i							
D13L	CPXV131	AAM13572			x	Rifampicin resistance protein	+
**Cats** j							
F12L	CPXV060	AAM13505			x	IEV-associated protein	+
**Rats** k							
B18R	CPXV019	AAM13466			x	Ankyrin-like protein	+
**Rabbits** e **and humans** i							
B20R	CPXV011	NP_619800		x	x	Ankyrin-like protein	+
**Rabbits** e **and cats** j							
A4L	*WR-123*CPXV136	AAO89402AAM13577	x	x	x	39 kDa core protein	+

a Identified by serological screen of *EL-VACV-0.2k-0.7k*.

b Identified by serological screen of *EL-CPXV-0.2k-0.7k*.

c Identified by serological screen of *EL-CPXV-0.2k-3k*.

d Orthopoxvirus Immune Epitope Database (IEDB) hits (www.immuneepitope.org).

e Reactive in immunized rabbits (Lister strain).

f Reference genome for alignment VACV strain Western Reserve (GenBank acc. no. AY243312).

g Reference genome for alignment CPXV strain Brighton Red (GenBank acc. no. AF482758).

i Reactive in immunized humans (VIG).

j Reactive in CPXV-infected cats.

k Reactive in CPXV-infected rats.

### Identification of immunogenic CPXV proteins

*EL-CPXV-0.2k-0.7k* and *EL-CPXV-0.2k-3k* were serologically screened to identify immunoreactive proteins of CPXV. A wide range of immunogenic proteins could be identified by screening both EL using sera from different species (Table 1). Only those immunoreactive clones encoding a single protein are included in Table 1. Immunoreactive clones with inserts encoding multiple proteins were also identified. These are not listed in Table 1 but are provided below. Screening of *EL-CPXV-0.2k-3k* using serum from a CPXV-infected cat resulted in the identification of immunoreactive clones encoding two proteins B19/B20 (NCBI accession numbers: NP_619993/NP_619994). Screening of the same library using anti-CPXV rat serum led to immunoreactive clones encoding the protein combinations A41/A42 (NCBI accession numbers: NP_619960/NP_619961), A43/no homologue (NCBI accession numbers: NP_619962/NP_619963), and I4/I5 (NCBI accession numbers: NP_619870/NP_619871).

### OPV proteins with diverse functions are immunogenic

The first main discovery of our serological screening was that immunogenic proteins can have most diverse functions. Interestingly, a few enzymes including A18 (DNA helicase), A48 (Thymidylate kinase), E9 (DNA polymerase), and H6 (DNA topoisomerase type I) were found to be immunogenic. Moreover, several ankyrin-like proteins (M1, B18, and B20) were found to be immunoreactive. A number of immune evasion proteins including A53 (tumor necrosis factor receptor, CrmC), C23 (chemokine-binding protein), and E3 (double-stranded RNA-binding protein) were also found to be antigenic. According to their different functions, the detected immunogenic OPV proteins varied in their location within the virus, ranging from core proteins (A3, A4) to membrane proteins (B22), including structural proteins (M1, B18, B20) as well as enzymes. Interestingly, there was no trend toward recognition of antigens with early or late gene expression.

### Genes encoding immunoreactive proteins are widely dispersed on an OPV genome

**Figure 2:**
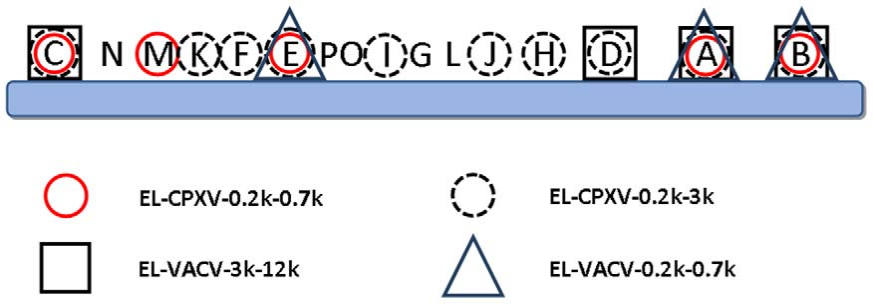
Genome-wide distribution of genes encoding immunogenic proteins. Shown is a generalized OPV genome map with HindIII restriction endonuclease DNA fragment letters (A-P). The icon-selected HindIII fragments encode at least one immunoreactive protein identified through plaque screening of the respective EL. Multiply-selected fragments encode immunoreactive proteins identified in more than one EL. The screenings were performed using sera from VACV -immunized and CPXV-infected humans and animals.

We also analyzed the distribution of the genes encoding immunoreactive proteins on a generalized OPV genome map (Figure 2). For simplicity, we again depicted an OPV genome containing HindIII restriction endonuclease DNA fragment letters (A-P). HindIII DNA fragments with genes encoding identified immunoreactive proteins are highlighted with a respective EL-specific icon. Figure 2 gives a detailed overview of the proportion of the OPV genome encoding immunogenic proteins. It contains the identified genes listed in Table 1 as well as the information on immunoreactive clones with inserts encoding two or more genes. The immunodominant genes were found to be part of nearly all different HindIII restriction fragments, including genes located in the central region of the genome as well as those in the terminal regions.

### VACV and CPXV cross-reactive proteins A4 and E3 are most antigenic

**Figure 3:**
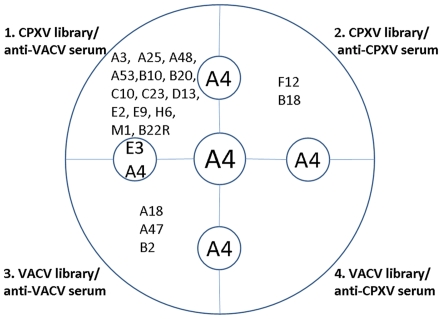
Determination of cross-reactive VACV and CPXV antigens. The main circle represents the entity of identified immunogenic OPV proteins. This set of proteins is divided into four subsets depending on the experimental setting: 1. CPXV EL screened with anti-VACV sera; 2. CPXV EL screened with anti-CPXV sera; 3. VACV EL screened with anti-VACV sera; and 4. VACV EL screened with anti-CPXV sera. Subsets 1 and 4 contain cross-reactive antigens. The inner circles represent the intersection of identified antigens. Thereby a differentiation is made between the same protein being present in two different subsets and a protein being present in all four subsets (central circle).

The constructed VACV and CPXV EL were also utilized to determine cross-reactive OPV antigens. For this purpose VACV EL were screened using anti-CPXV sera, and CPXV EL were screened using anti-VACV sera. To be able to identify cross-reactive proteins, the identified antigens were grouped into four subsets depending on the experimental conditions: 1. CPXV EL with anti-VACV sera, 2. CPXV libraries with anti-CPXV sera, 3. VACV libraries with anti-VACV sera, and 4. VACV libraries with anti-CPXV sera (Figure 3). All proteins attributed to subsets 1 and 4 represent cross-reactive antigens of VACV and CPXV. Moreover, the presence of a protein in more than one subset indicates a higher antigenicity. By determining the intersections of all subsets, protein A4 was found to be present in all four subsets. In addition, protein E3 was present in two of four subsets. We therefore conclude that out of the 16 proteins identified, A4 and E3 are the most antigenic cross-reactive proteins between CPXV and VACV.

## Discussion

Beside the discussion of potentially re-emerging VARV infections, the increasing number of CPXV infections, particularly in young people [13], [30]–[32], reawakened the interest in this zoonotic poxviral disease. As a result of discontinued smallpox vaccination in the younger generations, cowpox as well as monkeypox are regarded as emerging zoonotic hazards, requiring the development of new effective therapies or vaccines [17]. Recombinant subunit vaccines are considered to be safer than conventional live or attenuated vaccines [25]. Their development depends on the identification of cross-reactive OPV antigens that will likewise stimulate the most effective immune response to infectious virus. Many antigenic proteins of VACV have been identified as possible candidates in this context [33], [34]. However, genetic diversity between OPVs has not yet been taken into account [25] nor the fact that, beside VARV, monkeypox virus and CPXV are actually important pathogenic poxviruses.

In this study a set of 21 different immunogenic proteins could be identified by serological screening of genomic CPXV and VACV expression libraries (EL) using anti-VACV and anti-CPXV clinical sera. The whole set of these identified proteins was subsequently analyzed to identify cross-reactive OPV antigens.

The method described here allowed the identification of antibodies specific for OPV proteins. Like every method the serological screening of genomic λ-based EL has its benefits and limitations. One of the most important benefits is the fact that the construction of such a library does not require any prior knowledge of antibody targets. Therefore it is not necessary to predict ORFs of unknown proteins and to design primers using software, which allows the identification of new antigens. Furthermore, genomic EL ideally contain the entire host genome and express the complete set of virus-encoded proteins. This is especially advantageous for viruses with a large genome like that of the OPVs coding for up to 200 genes. For the libraries presented we have shown that they cover large parts of the viral genome by sequencing randomly picked clones. Therefore this approach allows the investigation of the humoral immune response without prior restriction to a certain protein class. Nevertheless, it is important to note that the fraction of sequences incorporated in a recombinant DNA library depends on the degree of partial DNA digestion [35]. The size of OPV proteins ranges from less than 50 amino acids to more than 1,000 amino acids, implying methodical problems. On the one hand, it is preferable to identify single OPV proteins during the screening process by using inserts of even shorter length. On the other hand, short inserts could result in the translation of incomplete proteins that lack the proper folding for obtaining the native immunogenic structure. To circumvent this and other limitations, EL containing inserts of different size were constructed. Moreover the serological screening of genomic EL is not an absolutely quantitative method but rather a qualitative approach. There is no guarantee that all immunoreactive proteins will be recognized and identified even when specific antibodies are present. However, due to the number of clones screened, several immunogenic proteins should be found. This constriction has been enhanced by pre-diluting the immune sera from 1/200 to 1/1,000. This elicited only the discovery of the most antigenic proteins that could be suitable for inclusion in vaccines.

Antigens expressed in *E. coli* may not completely parallel those expressed in OPV-infected eukaryotic cells, due to a lack of post-translational modifications. Additionally, solid-phase immobilization of library-expressed proteins may mask protein epitopes or affect the three-dimensional structure of the antigens. To assess the impact of these factors on the ensuing screening experiments, anti-rA27 antibodies were tested in ELISA assays. The anti-rA27 antibodies were generated in goats and mice immunized with a recombinant protein expressed in *E. coli*. The specificity of these antibodies was tested in ELISA by coating the recombinant protein as well as VACV particles. The antibodies were shown to recognize both. Thus, the immobilized recombinant A27 protein expressed in *E. coli* and coated onto plastic was still in a conformation similar to that present on virus particles. The same antibodies were used for the validation of the EL by an immunoscreening which resulted in the identification of the A27L gene. Additionally, the EL were screened with a monoclonal antibody raised against native CPXV particles. This screenings also resulted in immunoreactive plaques. Taken together these results demonstrate that phage-expressed proteins are detected by antibodies generated against native virus particles as well as by those raised against proteins expressed in *E. coli*.

Although a number of immunogenic OPV proteins has been identified so far, the cross-reactivity of these antigens was not yet adequately taken into account. Our primary goal was, therefore, to develop a method for the identification of proteins that are immunogenic and cross-reactive to different OPVs. For this purpose, VACV genome-containing EL were screened first with anti-VACV sera to demonstrate the suitability of the approach selected. Subsequently, the serological screening of EL containing VACV genome with anti-CPXV sera and vice versa was adopted to identify some of the cross-reactive proteins of VACV and CPXV.

Out of 21 identified immunogenic proteins, 16 were found to be cross-reactive. All 16 cross-reactive proteins could be identified through the screening combination of the CPXV EL with sera from VACV-immunized individuals, including an anti-VACV hyperimmune rabbit serum. Hyperimmunization is a vaccination method by which the same antigen is repeatedly administered, leading to boostered immune responses. On the other hand, the screening of a VACV EL using anti-CPXV clinical serum samples resulted in the identification of only one protein, A4. The different number of phage recombinants identified through the four different screening combinations has various reasons.

First of all, only immunoreactive clones with inserts encoding single proteins are listed in Table 1 and included in Figure 3. Thus, the screening results of *EL-VACV-3k-12k* which contains longer inserts are not depicted. The screening of *EL-CPXV-0.2k-3k* resulted in immunoreactive clones with inserts coding for single as well as multiple proteins. On the other hand, all clinical serum samples were obtained after a primary, naturally acquired CPXV infection. Here it is important to note that the serological screening of the EL with hyperimmune sera resulted in the identification of more cross-reactive antigens than a screening with a serum from naturally infected individuals did. These results perfectly correlate with the progression of a humoral immune response. Primary immune responses are often weak due to a limited amount of presented antigen. Therefore, only B cell clones producing high affinity antibodies or those with antigen receptor specificities for the most abundant antigen are selected for proliferation. Interestingly, A4 was identified as the most abundant protein in intracellular mature virions of VACV [3]. Following the second and subsequent boosts to the antigen, the affinity and amount of antibodies increase due to a process called affinity maturation. Hence, more antigenic determinants can be identified using conventional serological techniques. Therefore, clinical sera will usually be able to recognize fewer proteins than hyperimmune sera do. Finally, as mentioned above, the serological screening of genomic EL is not an absolutely quantitative method.

To get a general overview of cross-reactive antigens, it is therefore advisable to screen EL with sera of immunized or even better hyperimmunized individuals. Antibodies present in those sera have higher affinities for their cognate antigen due to somatic hypermutation. This allows an easier identification of the antibody targets using serological screening methods. However, screening of EL using sera obtained after a primary infection can result in the identification of the most antigenic or the most abundant cross-reactive proteins.

Among the identified cross-reactive proteins A4 was the most frequently identified immunogen. A4 (p39) was first identified by Maa and Esteban [36] as a highly antigenic protein eliciting a strong humoral immune response in rabbits and mice. They could also demonstrate the protein to be cross-reactive between VACV and CPXV and therefore supposed that it might have important biological functions [36]. More recently, DNA plasmids encoding the A4L gene were used to prime mice before a boost with VACV, resulting in an improvement of the immune reaction compared to the current vaccination strategy [23].

The immune evasion protein E3 was the second most frequently identified cross-reactive protein. E3 is an important virulence gene responsible for providing interferon resistance. E3 deletion mutants were shown to be less pathogenic in mice models [37]–[39], and thus it was proposed to use them as attenuated vaccines [37].

Among the identified proteins, D13, E3, A3, A4, H6, B2, and E2 were earlier shown to be immunogenic by using a VACV proteome microarray approach [34], [40]. Sahin and colleagues showed the proteins A4, A25, E9, F12, B2, and D13 to be antigenic by screening genomic shot-gun EL [41]. Finally, the immunogenicity of D13 and A4 was shown in a microELISA format with proteins derived from a mammalian *in vitro* expression system [33]. Thus, 10 out of 21 immunogenic proteins identified have been described as OPV antigens before. To our knowledge, the remaining 11 proteins have not been identified before as antibody targets using genome-wide screening approaches. We further compared our set of identified immunogenic proteins with the records of the Immune Epitope Database. This database provides a compilation of experimentally determined B- and T-cell epitopes [42]. For nearly all of the identified immunoreactive proteins, the presence of one or more Immune Epitope Database hits could be confirmed. However, to our knowledge, vaccinia homologues A53 (CPXV191), B22 (CPXV219), and C23 (CPXV003) are not yet known to contain any B- or T-cell epitopes. All of these proteins were also identified as being cross-reactive between VACV and CPXV. Thus, we were able to identify three new immunogenic OPV proteins which are cross-reactive between CPXV and VACV.

The screening of the EL using sera from different species (rabbits, humans, cats, and rats) resulted in the identification of two common antibody target proteins. The protein B20 was detected by antibodies present in VACV-immunized humans and rabbits. Furthermore, rabbit anti-VACV sera and cat anti-CPXV sera reacted with the protein A4. These commonly recognized antibody targets could be particularly well suited for vaccine design. However, the pooling of screening results for different species infected with different OPV strains could also result in conflicting data. It is therefore important to note that the type of infection and thus the protective immunity mounted against an OPV infection can depend on multiple factors. These include the species of OPV, the route of virus entry, as well as the genus/species of the host and its immune status [1], [43]. Furthermore, the impact of the primary versus secondary immune response to the obtained antigens should be taken into account [43].

An ideal subunit vaccine should protect against different OPV species. This could be achieved by including proteins which are highly conserved among OPV species and are therefore cross-protective. It has been shown that even slight heterogeneity of proteins could result in the loss of cross-protection [44], [45]. Out of 16 cross-reactive proteins identified, six (A3, A4, D13, E2, E9, and H6) are perfectly conserved in all members of the subfamily *Chordopoxvirinae* [11], [46] which also includes the genus OPV.

In summary, we have identified 16 cross-reactive proteins of CPXV and VACV by serological screening of genomic EL. Six of these proteins are perfectly conserved among all OPV species. Furthermore, we have identified three unknown immunogenic OPV proteins which are also cross-reactive between CPXV and VACV. Due to their conservation and frequency of detection, we propose that seven of the cross-reactive antigens identified, namely A3, A4, D13, E2, E3, E9, and H6, could be considered for inclusion in subunit vaccines. This could result in protection not only against CPXV but also against VARV and monkeypox virus. To our knowledge, only the protein A4 has so far been tested as a possible subunit vaccine component. The construction and serological screening of further genomic EL with VARV and monkeypox virus genomes could reveal more cross-reactive antigens and speed up the development of safer vaccines.

## Materials and Methods

### Enzymes

DNA restriction endonuclease Bsp143I and T4 DNA Ligase were purchased from Fermentas (St. Leon-Rot, Germany).

### Bacterial strains

The *E. coli* strains XL1-Blue MRF' and XLOLR were purchased from Agilent Technologies, Inc. (Santa Clara, CA, USA). The E. coli strain Rosetta™ was purchased from Novagen Inc. (Darmstadt, Germany).

### OPV strains

VACV strain New York City Board of Health (NYCBH; VR-1536™) Laboratories was purchased from American Type Culture Collection (ATCC). CPXV strain GuWi was isolated from a CPXV-infected elephant infected by a rat [47]. A CPXV named calpox virus was isolated from New World monkeys [48], [49].

### Recombinant A27 protein (rA27)

For the expression of A27, viral DNA was isolated from the calpox virus [48], [49]. The A27L gene was PCR amplified in a 50 µl reaction containing 1×PCR Buffer (Invitrogen, Darmstadt, Germany), 4 mM MgCl_2_, 100 µM dNTPs, 0.3 µM of each primer (CTgTACTTTCCATggACggAACTCTTTTCC and TTgAgTCTgCAgATATggTCgCCgTCCAgT), 1 unit Platinum Taq DNA polymerase (Invitrogen), and about 50 ng of template DNA. The cycling was carried out in a Mastercycler® ep gradient (Eppendorf, Hamburg, Germany) under the following conditions: 94°C for 2 min, followed by 30 cycles of 94°C for 20 sec, 63°C for 20 sec, and 72°C for 30 sec, and completed by 72°C for 10 min. The amplicons were purified using Qiaquick PCR purification Kit (Qiagen GmbH, Hilden, Germany) according to manufacturer's instructions. For expression of A27L gene, the purified amplicons were ligated into the pTriEx-3 vector (Novagen Inc.). The amplicons as well as the vector were predigested with the restriction enzymes NcoI and PstI prior to ligation reaction. The ligated DNA was subsequently used to transform competent *E. coli* strain Rosetta™ cells. The recombinant His-tag protein was finally purified under denaturing conditions using Protino®Ni-IDA columns (Macherey-Nagel, Düren, Germany).

### Sera, monoclonal antibodies, and enzyme conjugates

Vaccinia immune globulin (VIG), which is the immunoglobulin fraction of pooled vaccinia-hyperimmune human serum, was a generous gift from BEI Resources (Manassas, VA, USA). The hyperimmune rabbit anti-VACV (strain Lister) serum was obtained from Acris Antibodies GmbH (Herford, Germany). The clinical serum samples included human, cat, and rat sera collected from CPXV-infected individuals. The human serum was collected 8–9 weeks post infection, the cat serum 3–4 weeks post infection, and the rat serum 2–3 weeks post infection. All clinical material was provided by the German Consultant Laboratory for Poxvirus infections (Robert Koch Institute, Berlin, Germany).

The human anti-dengue virus serum was collected 2–3 weeks post infection. To show the absence of anti-OPV antibodies, both the human anti-dengue virus serum and the human poxvirus-naive serum were tested by immunofluorescence assay, as described elsewhere [49]. Briefly, for the detection of OPV-specific antibodies slides were coated with CPXV GuWi-infected Hep2 cells. For the detection of dengue virus-specific antibodies slides were coated with dengue virus-infected (dengue virus type 1) Vero E6 cells (European Collection of Cell Cultures, ECACC: 85020205). The polyclonal goat anti-rA27 serum was generated by subcutaneously immunizing a goat with approximately 200 µg of the recombinant A27 protein expressed in *E. coli*. TiterMax® Gold was used as adjuvant. The goat was boosted once subcutaneously four months later with about 300 µg antigen and the serum was collected two weeks thereafter.

The monoclonal anti-CPXV 3D11 antibody [29] was kindly provided by Claus Peter Czerny. The monoclonal mouse anti-rA27 A1/6-15 was generated using the hybridoma technology by immunizing C57BL/6 mice with the recombinant A27 protein expressed in *E. coli*.

Horseradish peroxidase (HRP)-conjugated goat anti-human IgG (gamma chain) and peroxidase-rec protein A were purchased from Invitrogen (Darmstadt, Germany). Goat anti-rabbit IgG-HRP and Goat anti-mouse IgG Fcγ-HRP were obtained from Jackson Immunoresearch (West Grove, PA, USA). Donkey anti-goat IgG (H+L)-HRP was purchased from Dianova GmbH (Hamburg, Germany).

### ELISA assays

For ELISA testing of anti-rA27 antibodies 200 ng of rA27 or 5×10^6^ UV-inactivated VACV particles (strain NYCBH) were coated over night in 100 µl/well (0.1 M NaHCO_3_ pH 9.6) of a 96-well MaxiSorp ELISA plate (Nunc, Langenselbold, Germany). The wells were then blocked with Tris-buffered saline containing 0.05% Tween 20 (TBS-T) and 3% BSA for 1 hr at room temperature. After four washing steps (300 µl/well TBS-T) the diluted anti-rA27 antibodies (in TBS-T with 0.25% BSA) were added at 100 µl/well and incubated for 1 hr at room temperature. Subsequently, the wells were washed again four times and the diluted HRP-conjugated goat anti-mouse or donkey anti-goat antibodies were added (100 µl/well, 1∶5,000 diluted in TBS-T with 0.25% BSA) and incubated for 1 hr at room temperature. After four further washes, bound HRP was detected using 3,3′,5,5′-Tetramethylbenzidine (TMB) substrate tablets (Sigma, St. Louis, MO, USA) and assayed at 450 nm with a reference measurement at 620 nm (Infinite®200 PRO microplate reader, Tecan Group Ltd., Männedorf, Switzerland).

### Sera pre-treatment

Sera generally contain antibodies to *E. coli* proteins, which can cause a high background signal. To reduce this background signal, sera were pre-incubated with *E. coli* (strain XL1-Blue MRF′) lysate. The lysate was prepared by growing an *E. coli* culture to saturation and harvesting the cells by centrifugation (15 min, 1,500×g). The cells were then resuspended in a buffer containing 50 mM Tris-HCl (pH 8.0) and 10 mM EDTA and were subsequently broken by three successive freeze-thaw cycles, followed by 3×1 min sonication steps (Sonifier S-450D, Branson, Danbury, CT, USA). The cell debris was then removed by another centrifugation step (15 min, 1,500×g). The resulting lysate (supernatant) was used for incubation with the diluted serum for 30 min at room temperature.

### Ethics statement

The clinical cat and rat sera used to screen the genomic expression libraries were provided by the German Consultant Laboratory for Poxvirus infections (Robert Koch Institute, Berlin, Germany). No specific ethical approval was required, since all sera were diagnostic material and no animal experiments using laboratory animals were performed.

### Preparation of genomic DNA for cloning

#### Purification of genomic DNA

OPVs were grown in 175 cm^2^ culture flasks containing a monolayer of Hep2 cells (ATCC) for three days (VACV) or five days (CPXV), respectively. Approximately 2.7×10^7^ cells were infected with a virus input multiplicity of infection (MOI) of 0.25 (VACV) and 0.1 (CPXV), respectively. The preparation of poxvirus DNA from the cytoplasm of infected Hep2 cells was performed as described in [50]. The concentration of the prepared DNA was determined using NanoDrop 1000 (Thermo Scientific, Wilmington, DE, USA). The integrity of the genomic DNA was estimated on a 0.5% agarose gel prepared in 1×TAE buffer with 1 µg/ml ethidium bromide.

#### Real-time PCR

The ratio between poxvirus and human DNA was estimated by real-time PCR using two different assays. For the detection of OPV DNA the OPV assay was used. The cellular DNA was detected by using the c-myc assay which detects the housekeeping gene c-myc. The real-time PCR was performed as described elsewhere [51]. As quantitative calibration standards plasmids with the respective target sequence were measured in each run. For plasmid construction the corresponding sequences were amplified and cloned into a TOPO TA vector (Invitrogen, Karlsruhe, Germany) as described previously [52]. The cycling was carried out in an Mx3000P QPCR system (Agilent Technologies, Inc.) under the following conditions: 95°C for 10 min, followed by 40 cycles of 95°C for 15 sec and 60°C for 30 sec.

#### Partial digestion of genomic DNA

Partial digestion of genomic poxvirus DNA was performed using the DNA restriction endonuclease Bsp143I (Fermentas). The amount of Bsp143I applied depended on the DNA insert size desired for cloning. For a fragment size of 3 kb to 12 kb a BspI concentration of 0.01 U/µg to digest about 60 µg of genomic poxvirus DNA was convenient. A fragment size of 0.2 kb to 3 kb could be achieved by digesting about 50 µg DNA with 0.15 U/µg of Bsp143I. For a fragment size of 0.2 kb to 0.7 kb, 0.5 U/µg of Bsp143I for about 50 µg DNA was convenient.

#### Size fractionation

The partially digested poxvirus DNA was fractionated on agarose gels at 50 V for 2–3 hr. The agarose concentration depended on the fragment size to be fractionated: 0.5% for 3 kb to 12 kb fragments, 0.8% for 0.2 kb to 3 kb fragments, and 1.5% for 0.2 kb to 0.7 kb. The fractionated DNA was visualized under long-wave UV light, and the desired fragment range was excised using a scalpel. The excised gel slices were weighed and the DNA extracted using Nucleospin Extract II Kit (MachereyNagel) according to the manufacturer's instructions. The extracted DNA was diluted in 15 µl elution buffer and the DNA concentration determined using NanoDrop 1000.

### Construction of OPV expression libraries

Starting from ligation reaction, all genomic ELs were constructed using the “ZAP Express® Predigested Gigapack® III Gold Cloning Kit” (purchased from Stratagene, La Jolla, CA, USA, now Agilent Technologies, Inc.) with the BamHI/CIAP-treated vector (former catalog #239615, currently not available) according to manufacturer's instructions. Methods for which modifications to the original protocol or amount specifications of reagents were necessary are described below.

The partial digestion of DNA with Bsp143I enzyme results in DNA ends compatible with those produced by the restriction enzyme BamHI. Thus, the partially digested genomic OPV DNA was directly ligated into the BamHI-predigested ZAP Express vector without modifying the DNA ends. The following DNA amounts were ligated for each EL: 336 ng for *EL-VACV-3k-12k*, 185 ng for *EL-VACV-0.2k-0.7k*, 80 ng for *EL-CPXV-0.2k-0.7k*, and 36 ng for *EL-CPXV-0.2k-3k* in a final volume of 7 µl. DNA from ligation reactions was directly used for the packaging reactions. The following volumes of ligation reaction were used for packaging: 3.5 µl for *EL-VACV-3k-12k*, 4 µl for *EL-VACV-0.2k-0.7k* and *EL-CPXV-0.2k-0.7k*, and 7 µl for *EL-CPXV-0.2k-3k*.

### Validation of OPV expression libraries

For an assessment of their quality all four EL constructed were extensively validated. Hereby, the representativeness of the EL was assessed by randomly picking 25–60 recombinant plaques from every library and by sequencing the DNA inserts. The obtained sequences were aligned to a reference poxvirus genome to estimate the distribution of the sequences. Additionally, all four EL were serologically screened using monoclonal (anti-rA27 A1/6-15 anti-CPXV 3D11) and polyclonal (goat anti-rA27) antibodies.

### Plaque immunoscreening of OPV expression libraries

All four constructed OPV EL were screened with selected polyclonal and monoclonal antibodies. The plaque-screening procedure was performed as described in the picoBlue™ Immunoscreening Kit (Agilent Technologies, Inc.) with minor changes. Briefly, 150 mm dishes with phage plaques growing for 4 hr at 42°C on a lawn of *E. coli* XL-1 Blue MRF′ cells in 0.7% soft agar were overlaid with nitrocellulose filter disks (PALL Gelman Laboratory, Ann Arbor, MI, USA) presoaked in 10 mM isopropyl-β-thiogalactoside (IPTG). After incubation for 4 hr at 37°C, the filters were removed, washed in TBS-T at room temperature, and blocked in Tris-buffered saline (TBS) containing 1% bovine serum albumin. Filters were then incubated in succession with monoclonal antibodies/sera and HRP-conjugated secondary antibodies, with 3–5 TBS-T washes between every incubation step. The monoclonal antibodies were diluted to 0.5 µg/ml in TBS with 1% BSA. The sera VIG, rabbit anti-VACV, cat anti-CPXV, and goat anti-rA27 were diluted 1∶1,000. Rat anti-CPXV serum was diluted 1∶200. The binding of HRP-conjugated antibodies was detected through the color development reaction with the substrate 3-Amino-9-ethylcarbazole (AEC, Sigma). Immunopositive plaques resulted in small reddish dots on the membrane. This membrane was then aligned with the original agar plate, allowing identification of corresponding plaques. After staining, positive plaques were picked, suspended in buffer, and plaque-purified once on 90 mm dishes. For this, phage solutions were diluted (10^−1^–10^−3^), plated, and incubated over night at 37°C. The plates were again overlaid with nitrocellulose membranes (PALL Gelman Laboratory), incubated for 4 hr at 37°C, and stained. Positive plaques were picked again (without adding chloroform to buffer), and the contained pBK-CMV phagemid vector was excised *in vivo* as described in the “ZAP Express® Predigested Gigapack® III Gold Cloning Kit” (Agilent Technologies, Inc.) instruction manual. From the bacterial colonies appearing on the agar plates the excised pBK-CMV double-stranded phagemid vectors were isolated using NucleoSpin® Plasmid QuickPure Kit (Macherey-Nagel) according to the manufacturer's instructions and the DNA amount quantified using the NanoDrop1000.

### PCR and DNA sequencing

The isolated pBK-CMV phagemid vectors were used as templates for PCR. For the amplification of long inserts (≥3 kb) the “Expand Long Range, dNTPack” (Roche Diagnostics GmbH, Mannheim, Germany) was used according to the manufacturer's instructions as a 25 µl reaction containing 3% DMSO. The amplification of shorter fragments was performed as a 25 µl reaction containing 1×PCR Buffer (Invitrogen), 4 mM MgCl_2_, 100 µM dNTPs, 0.3 µM of each primer (T7 primer: gTAATACgACTCACTATAgggCg and T3 primer: ATTAACCCTCACTAAAgggA), 1 unit of Platinum Taq DNA polymerase (Invitrogen), and about 50 ng of template DNA. The cycling was carried out in a Mastercycler ep gradient (Eppendorf) under the following conditions: 95°C for 5 min, followed by 40 cycles of 95°C for 30 s, 57°C for 30 s, and 72°C for 60 s, completed by 72°C for 5 min, and cooling down to 4°C until further processing. The amplified DNA inserts were cycle sequenced using the flanking primers T3 and T7 and the BigDye® Terminators chemistry (Applied Biosystems, Weiterstadt, Germany).

### Data processing

The obtained sequences were aligned to VACV strain Western Reserve (WR) genome (GenBank acc. no. AY243312.1) for VACV genome-containing EL and to CPXV strain Brighton Red (GenBank acc. no. AF482758) for CPXV libraries. The alignment was performed using the BLASTN program by optimizing for highly similar sequences (megablast).

## Supporting Information

Figure S1 **Validation of constructed genomic OPV expression libraries.** Recombinant plaques were picked, the insert DNA sequenced and the obtained sequence aligned to a reference genome. Shown is the distribution of DNA-inserts obtained from recombinant clones from (A) *EL-VACV-3k-12k* displayed on a VACV genome (GenBank AY243312.1), and (B) *EL-CPXV-0.2k-0.7k* displayed on a CPXV genome (GenBank AF482758.2). The graphics were created using the nucleotide database on the NCBI website (http://www.ncbi.nlm.nih.gov/nuccore). By choosing the appropriate reference genome and the display setting “graphics”, the DNA inserts from recombinant clones could be defined as markers. Each DNA insert obtained from a recombinant clone is represented by a dot and the respective color-shaded bar. These colored bars indicate the size of the DNA insert and the covered genes. The pairs of horizontal green and red bars represent annotated genes on the dsDNA poxvirus genome.
(TIF)

Figure S2 **Screening of an expression library with an anti-CPXV antibody.** For a validation of their complexity the constructed genomic EL were serologically screened with the monoclonal antibody 3D11 that was raised against native CPXV particles. Immunopositive signals are exemplary indicated through white arrows on a stained nitrocellulose filter obtained through screening of *EL-VACV-3k-12k*.
(TIF)

Figure S3 **Validation of anti-rA27 antibody reactivity in ELISA.** Polyclonal goat anti-rA27 and monoclonal mouse anti-rA27 were generated by immunizing with a recombinant A27 protein expressed in E. coli. The target reactivities of these antibodies were tested in an ELISA by coating the recombinant antigen and whole VACV particles onto ELISA plates and incubating with serially diluted antibodies: (A) Goat anti-rA27 serum, (B) Mouse anti-rA27 monoclonal antibody.
(TIF)
