# Supplementary figures and images for: Genomic Expression Libraries for the Identification of Cross-Reactive Orthopoxvirus Antigens
Source: PLoS One. 2011 Jul 14;6(7):e21950. doi: 10.1371/journal.pone.0021950 (PMC3136487; doi:10.1371/journal.pone.0021950)

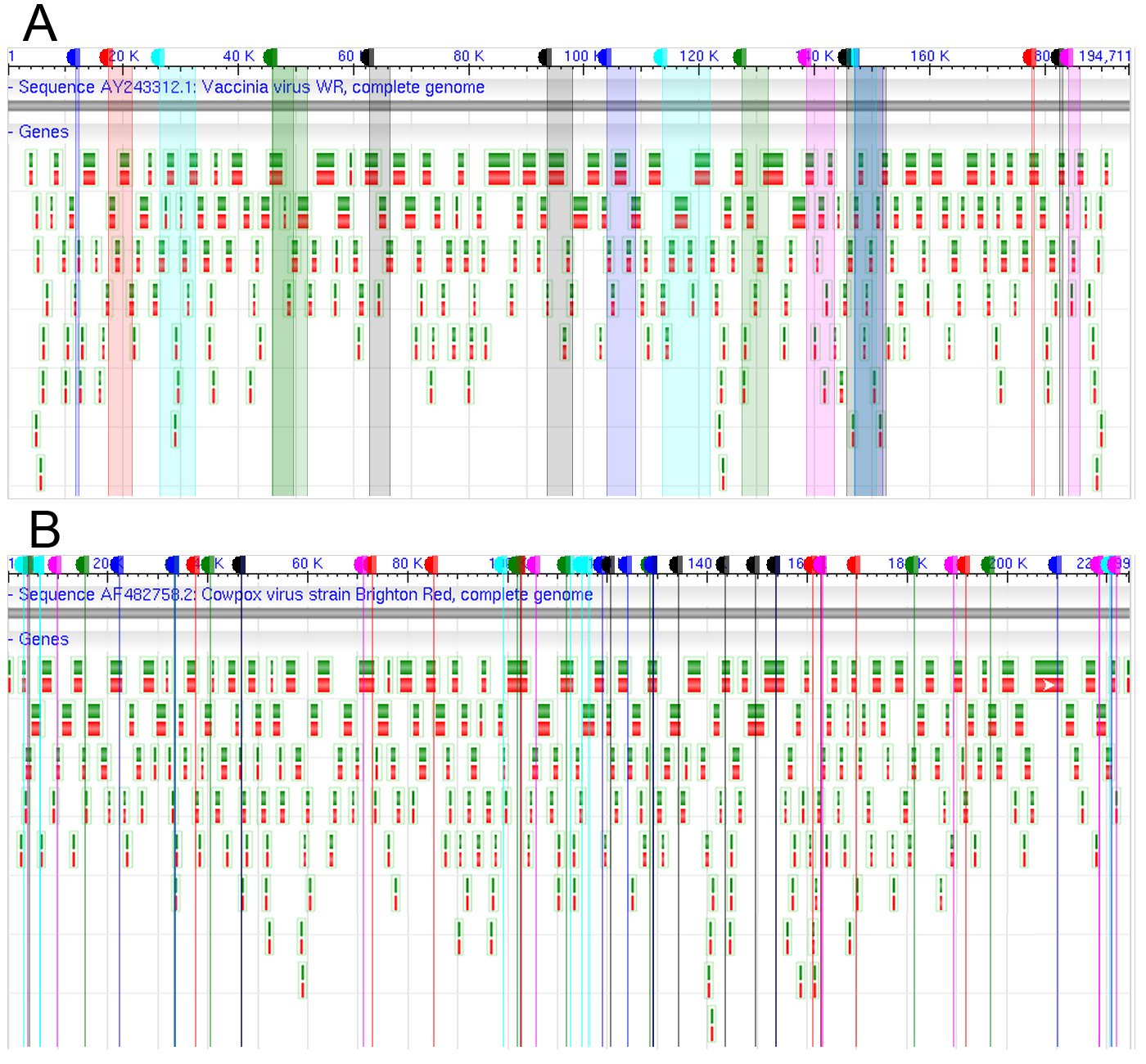

Supplement: Figure S1 — Validation of constructed genomic OPV expression libraries. Recombinant plaques were picked, the insert DNA sequenced and the obtained sequence aligned to a reference genome. Shown is the distribution of DNA-inserts obtained from recombinant clones from (A) EL-VACV-3k-12k displayed on a VACV genome (GenBank AY243312.1), and (B) EL-CPXV-0.2k-0.7k displayed on a CPXV genome (GenBank AF482758.2). The graphics were created using the nucleotide database on the NCBI website (http://www.ncbi.nlm.nih.gov/nuccore). By choosing the appropriate reference genome and the display setting “graphics”, the DNA inserts from recombinant clones could be defined as markers. Each DNA insert obtained from a recombinant clone is represented by a dot and the respective color-shaded bar. These colored bars indicate the size of the DNA insert and the covered genes. The pairs of horizontal green and red bars represent annotated genes on the dsDNA poxvirus genome. (TIF) [file pone.0021950.s001.tif]

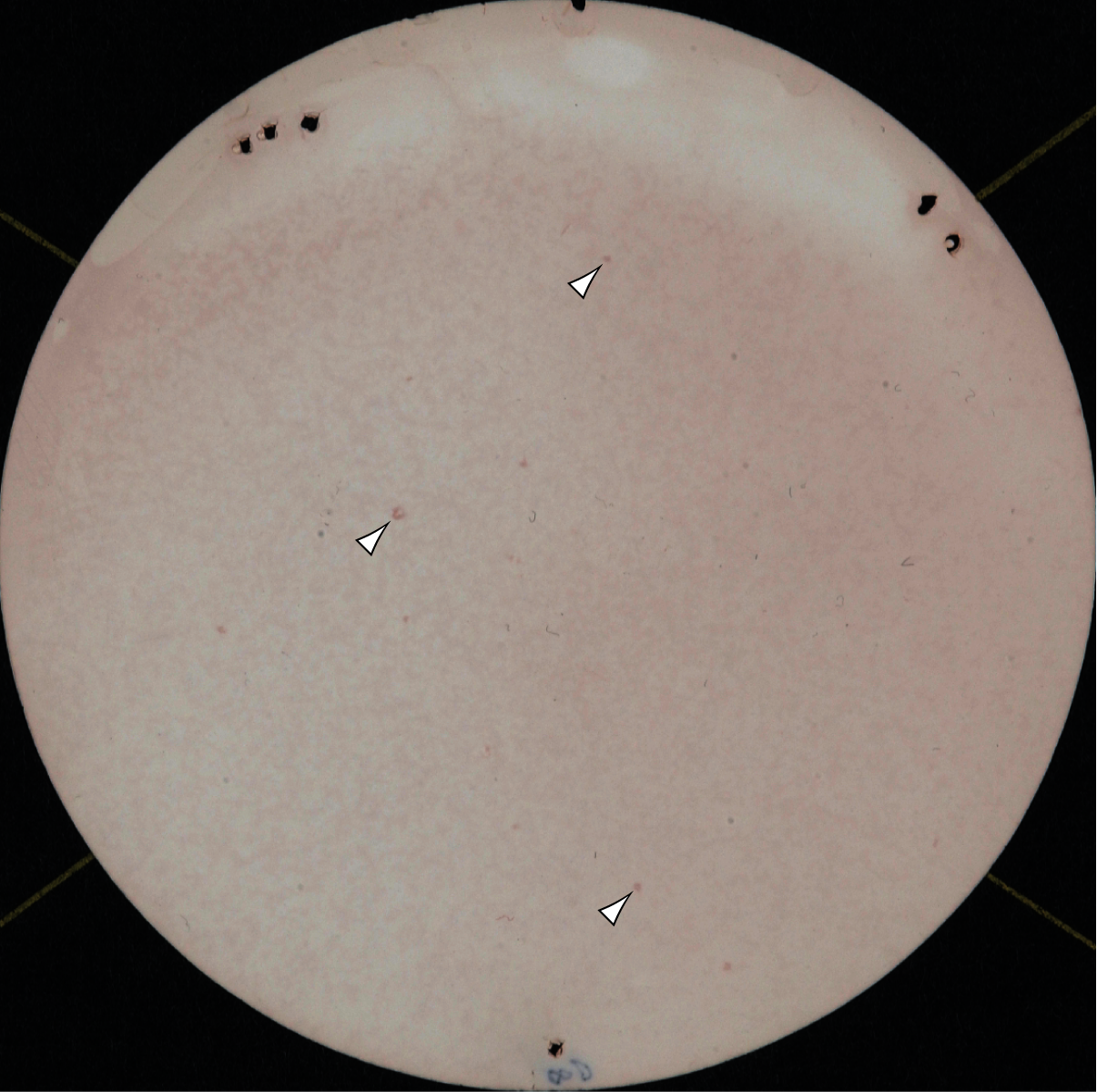

Supplement: Figure S2 — Screening of an expression library with an anti-CPXV antibody. For a validation of their complexity the constructed genomic EL were serologically screened with the monoclonal antibody 3D11 that was raised against native CPXV particles. Immunopositive signals are exemplary indicated through white arrows on a stained nitrocellulose filter obtained through screening of EL-VACV-3k-12k. (TIF) [file pone.0021950.s002.tif]

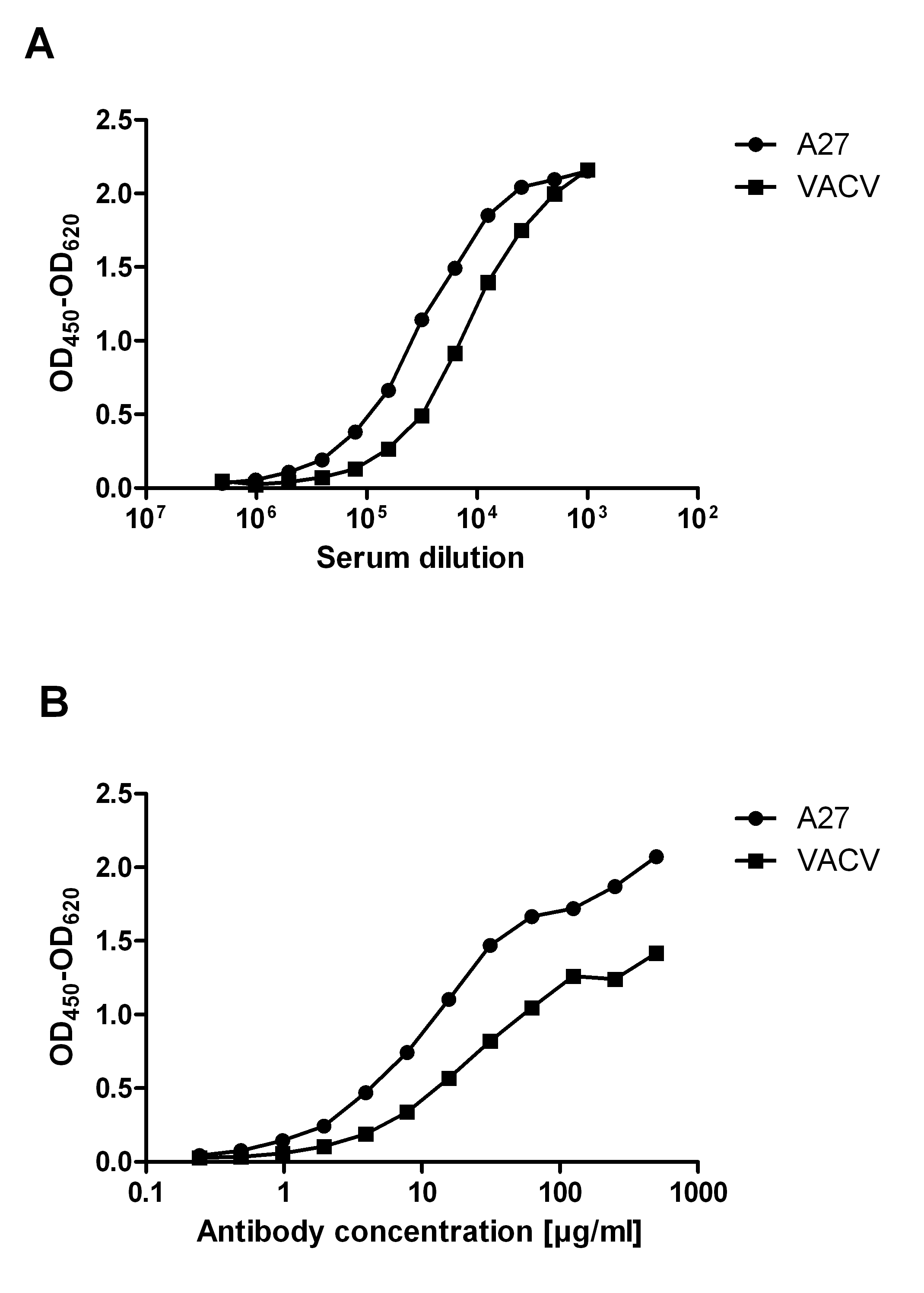

Supplement: Figure S3 — Validation of anti-rA27 antibody reactivity in ELISA. Polyclonal goat anti-rA27 and monoclonal mouse anti-rA27 were generated by immunizing with a recombinant A27 protein expressed in E. coli. The target reactivities of these antibodies were tested in an ELISA by coating the recombinant antigen and whole VACV particles onto ELISA plates and incubating with serially diluted antibodies: (A) Goat anti-rA27 serum, (B) Mouse anti-rA27 monoclonal antibody. (TIF) [file pone.0021950.s003.tif]
